# Functional Characterization of Fad Genes from Two Chemosymbiotic Bivalves Inhabiting the Haima Cold Seep

**DOI:** 10.3390/genes17060662

**Published:** 2026-06-05

**Authors:** Runlin Liu, Meixia Chen, Danli Jiang, Helu Liu

**Affiliations:** 1 NHC Key Laboratory of Tropical Disease Control, School of Tropical Medicine, Hainan Medical University, Haikou 571199, China; liurl@idsse.ac.cn; 2 Institute of Deep-Sea Science and Engineering, Chinese Academy of Sciences, Sanya 572000, China; chenmx@idsse.ac.cn; 3 International Center for Aging and Cancer, Hainan Medical University, Haikou 57119, China

**Keywords:** cold seep, chemosymbiotic bivalves, fatty acid desaturase, long-chain polyunsaturated fatty acids (LC-PUFA), non-methylene-interrupted (NMI) fatty acids, deep-sea adaptation

## Abstract

Background/Objectives: Chemosymbiotic bivalves play a fundamental role in deep-sea cold seep and hydrothermal vent ecosystems, where essential long-chain polyunsaturated fatty acids (LC-PUFAs) are scarce. Whether these bivalves retain the capacity for endogenous PUFA synthesis remains poorly understood. Here, we investigated the PUFA biosynthetic capacity of two dominant chemosymbiotic bivalves from the Haima cold seep—the mussel *Gigantidas haimaensis* and the clam *Archivesica marissinica*. Methods: Genome and transcriptome assembly revealed three fatty acid desaturase (*Fad*) genes per species, which phylogenetically clustered into Δ5 (*GhFads1*, *GhFads2*; *AmFads1*, *AmFads2*) and Δ6/8 (*GhFads3*, *AmFads3*) clades, with lineage-specific duplications within the Δ5 clade. Functional assays were performed in yeast to characterize the activity of these enzymes. Results:Both Fads3 enzymes (Δ6/8 clade) convert C20:3n-6 and C20:4n-3 into C20:4n-6 and C20:5n-3, respectively, exhibiting Δ8-desaturase activity. Notably, Δ5-clade isoforms showed divergent substrate specificities: *GhFads2* and *AmFads1* functioned as classical Δ5-desaturases on PUFA substrates, whereas *GhFads1* and *AmFads2* specifically desaturated the bacterial monounsaturated fatty acid (MUFA) C18:1n-7 to produce C18:2n PUFAs. Conclusions: Together, our results reveal that cold-seep bivalves retain endogenous LC-PUFA biosynthetic capacity and have evolved duplicated Δ5-desaturases with novel regioselectivity toward bacterial MUFAs, likely representing an adaptive membrane modification for survival under extreme deep-sea conditions.

## 1. Introduction

Polyunsaturated fatty acids (PUFAs), especially long-chain PUFAs (LC-PUFAs) such as arachidonic acid (ARA, 20:4n-6), eicosapentaenoic acid (EPA, 20:5n-3), and docosahexaenoic acid (DHA, 22:6n-3), are essential for animal growth, development, and physiological regulation [1,2]. In marine ecosystems, PUFAs are primarily synthesized by phytoplankton and other microorganisms (e.g., diatoms, cyanobacteria, oomycetes, and fungi), and are subsequently transferred through the food web [3,4]. Consequently, coastal animals are generally rich in PUFAs. In contrast, deep-sea chemosynthetic ecosystems, including hydrothermal vents and cold seeps, exhibit a distinct fatty acid profile. In these ecosystems, sulfur-oxidizing bacterial symbionts typically produce monounsaturated fatty acids (MUFAs) such as C16:1n-7 and C18:1n-7 [5], while aerobic methanotrophs synthesize n-8 and n-9 MUFAs [6]. PUFAs are not commonly synthesized by these chemosynthetic prokaryotes [5,6]. As a result, seep and vent-dwelling fauna often display low levels of essential PUFAs and instead accumulate bacterial MUFAs, which contrasts sharply with that of their shallow-water relatives.

Most animals lack the complete enzymatic machinery for *de novo* PUFA biosynthesis due to the absence of ω3 fatty acid desaturase (ω3 Fad), although ω3 *Fad* genes have been identified in several animal lineages [7,8]. Consequently, animals must obtain PUFAs either directly from their diet or indirectly through the desaturation and elongation of precursor PUFAs such as linoleic acid (LA) and α-linolenic acid (ALA). The fatty acid desaturase (Fad) gene family encodes enzymes that introduce double bonds into fatty acid substrates, with Δ5 and Δ6/8 desaturases playing particularly important roles in PUFA precursor desaturation. Previous studies using ^14^C-labeled fatty acids have confirmed that marine molluscs can modify their LC-PUFA profiles [9], and endogenous Fad genes have been widely investigated [10]. Functionally characterized *fad* genes have been isolated from several littoral molluscs, including *Octopus vulgaris* [11], *Haliotis discus* [12], *Chlamys nobilis* [13,14], *Sinonovacula constricta* [15], and *Mulinia lateralis* [16]. With the exception of a Δ8 desaturase from *C. nobilis* and a Δ6 desaturase from *S. constricta*, all functionally characterized molluscan Fads exhibit Δ5 desaturase activity. Chemosymbiotic bivalves play a foundational role in deep-sea vent and cold-seep ecosystems by harboring endosymbiotic bacteria that perform chemosynthesis. However, the phylogenetic relationships and functional diversity of the Fad gene family in vent/seep bivalves remain entirely unexplored.

Discovered in 2015, the Haima cold seep is one of only two known active seeps in the South China Sea, situated at depths of 1360–1400 m [17]. Characterized by high biomass and species richness, it hosts over 80 documented species and serves as an excellent model for seep ecology. Among the dominant taxa are the mussel *G. haimaensis* and the clam *A. marissinica*, both newly described species that form patchy beds and play foundational roles in the seep ecosystem through their symbiotic nutrition [17,18]. In recent years, publicly available genome and transcriptome data for Haima cold-seep fauna have increased substantially. This growing resource, together with advances in bioinformatics, has enabled the prediction and identification of functional genes across diverse species. In this study, we identified and characterized Fad genes from *G. haimaensis* and *A. marissinica*. We performed phylogenetic analyses to elucidate the evolution of their Fad gene family within bivalve and determined the functional activity of the encoded enzymes through heterologous expression in yeast.

## 2. Materials and Methods

### 2.1. Sample Collection

Samples were collected in October 2023 during cruise TS2-30 aboard the research vessel *TANSUOERHAO* at the Haima cold seep in the South China Sea, using the human-occupied vehicle (HOV) *SHENHAIYONGSHI*. Specimens were collected using the HOV’s mechanical arm and immediately placed into an insulated biobox to minimize thermal stress. Upon retrieval on deck, samples were flash-frozen in liquid nitrogen and subsequently stored at −80 °C until further processing. Species identification was confirmed by sequencing the mitochondrial cytochrome c oxidase subunit I (*CoxI*) gene using the universal primers LCO1490 and HCO2198 (Table 1). PCR amplification was carried out on an Applied Biosystems Veriti 96-Well thermal cycler using PrimeSTAR^®^ GXL Premix (Takara, Dalian, China, #R053A). The thermal cycling protocol consisted of an initial denaturation at 98 °C for 30 s, followed by 35 cycles of denaturation at 98 °C for 10 s, annealing at 55 °C for 5 s, and extension at 68 °C for 30 s.

### 2.2. Fad Identification and Phylogenetic Analyses

Transcriptomic data for *G. haimaensis* were obtained from the Science Data Bank (https://www.scidb.cn/anonymous/Wk5Cbm1t) on 5 March 2025; its assembled genomic data were retrieved from the NCBI Genome database under accession number GCA_054130545. Transcriptomic data for *A. marissinica* were retrieved from the NCBI Sequence Read Archive under access no. SRP259750, and its assembled genomic data were obtained from NCBI Genome under accession number GCA_014843695. Raw transcriptomic reads were quality-assessed with FastQC and preprocessed (quality trimming and adapter removal) using fastp v0.12.4. Clean reads were *de novo* assembled with Trinity v2.5.1 under default parameters. Redundant transcripts showing ≥95% sequence similarity were clustered using CD-HIT-EST v4.6.8. Open reading frames (ORFs) and corresponding putative peptide sequences were predicted from the non-redundant transcript sets using TransDecoder v5.7.1. Candidate fatty acid desaturase (Fad) genes were identified by performing homology searches against both the predicted peptide sequences and the assembled genomes with BLASTP v2.12.0. Multiple sequence alignments of the putative Fad protein sequences were generated using MUSCLE as implemented in MEGA v.11 [19]. A neighbor-joining (NJ) phylogenetic tree was constructed with the same software based on the aligned sequences. Branch support was assessed using bootstrap analysis with 1000 replicates under the Poisson Model.

### 2.3. RNA Extraction

Total RNA was isolated from adductor tissues of the mussel *G. haimaensis* and the clam *Ar. marissinica* using a TRIzol-based method. Approximately 100 mg of frozen tissue was homogenized in 1 mL of QIAzol Lysis Reagent (Qiagen, Hilden, Germany, #79306). Chloroform (200 µL) was then added, and the mixture was vortexed vigorously for 15 s, incubated at room temperature for 2–3 min, and centrifuged at 12,000× *g* for 15 min at 4 °C. The upper aqueous phase was carefully transferred to a fresh 1.5 mL microcentrifuge tube and mixed with an equal volume of isopropanol. After incubation at room temperature for 10 min, RNA was precipitated by centrifugation at 12,000× *g* for 10 min at 4 °C. The supernatant was discarded, and the RNA pellet was washed once with 1 mL of 75% ethanol, followed by centrifugation at 7500× *g* for 5 min at 4 °C. The ethanol was removed, and the pellet was air-dried briefly at room temperature before resuspension in RNase-free water. RNA concentration and purity were determined using a NanoDrop 2000 spectrophotometer (Thermofisher Scientific, Waltham, MA, USA), and RNA integrity was verified by 1% agarose gel electrophoresis.

### 2.4. cDNA Synthesis and qPCR Expression

First-strand cDNA was synthesized from 1 µg of total RNA using the RevertAid First Strand cDNA Synthesis Kit (Thermofisher Scientific, Waltham, MA, USA, #K1622) according to the manufacturer’s protocol. Briefly, total RNA was mixed with oligo(dT)_18_ primers and reaction buffer provided with the kit, denatured at 65 °C for 5 min, and immediately chilled on ice. dNTP mix, RNase inhibitor, and RevertAid Reverse Transcriptase were then added. The reverse transcription reaction was carried out at 42 °C for 60 min, followed by enzyme inactivation at 70 °C for 5 min. Then the synthesized cDNA was stored at −20 °C until further use.

Quantitative real-time PCR (qPCR) was performed on an Applied Biosystems 7500 Fast Real-Time PCR System. Each 20 µL reaction contained 1× Universal SYBR Green Master (Roche, Indianapolis, IN, USA, #04913914001), gene-specific primers (Table 1), and cDNA template. The thermal cycling conditions were as follows: initial denaturation at 95 °C for 30 s, followed by 40 cycles of 95 °C for 10 s and 60 °C for 30 s. Six individuals from each group were used in the qPCR assay, and all reactions were performed in triplicate. A melt curve analysis was conducted post-amplification to confirm primer specificity. The relative expression of target genes was calculated using the comparative 2^−ΔΔCt^ method, with normalization to the endogenous reference gene *gapdh*.

### 2.5. Plasmid Construction

Open reading frames (ORFs) encoding candidate Fads were amplified from cDNA using PrimeSTAR^®^ GXL Premix (Takara, Dalian, China, #R053A). PCR reactions consisted of an initial denaturation at 98 °C for 30 s, followed by 35 cycles of 98 °C for 10 s, annealing at 55 °C for 5 s, and extension at 68 °C for 60 s. Primers containing the respective restriction sites are listed in Table 1. Amplified fragments were purified, digested with the indicated restriction enzymes, and ligated into the same digested yeast expression vector pYES2 (Invitrogen, Carlsbad, CA, USA, #V82520). Recombinant plasmids were transformed into Competent Cell *Escherichia coli* DH5α and verified by Sanger sequencing.

### 2.6. Heterologous Expression in Yeast

Sequence-confirmed plasmids were transformed into *Saccharomyces cerevisiae* strain INVSc1 (Invitrogen, CA, USA) following the manufacturer’s protocol for the pYES2 vector. Transformants were selected on synthetic complete minimal medium lacking uracil (SC-U). For functional expression, recombinant yeast strains were cultured in SC-U broth containing 2% galactose to induce expression from the GAL1 promoter, as described previously [20]. Exogenous fatty acid substrates (C18:2n-6, C18:3n-3, C20:2n-6, C20:3n-3, C20:3n-6, and C18:1n-7) were saponified in 0.5 M KOH-ethanol and added to the culture medium at a final concentration of 0.75 µM. All fatty acid substrates were purchased from Cayman Chemical (Ann Arbor, MI, USA). Yeast cells were harvested by centrifugation, and total lipids were extracted by homogenization in chloroform:methanol (2:1, *v*/*v*) containing 0.01% BHT. Fatty acid methyl esters (FAMEs) were prepared from the extracted lipids, recovered by hexane extraction, and analyzed by gas chromatography as described above. The proportion of substrate fatty acid converted to desaturation product was calculated from the gas chromatograms as 100 × [product area/(product area + substrate area)].

### 2.7. Fatty Acid Methyl Ester (FAME) Analysis

FAMEs were analyzed by gas chromatography using an Agilent 6890 GC equipped with a flame ionization detector and an HP-88 capillary column (60 m × 0.25 mm i.d., 0.25 µm film thickness; Agilent, Santa Clara, CA, USA), and time-of-flight mass spectrometry (Agilent 8890-LECO Pegasus BT series, MI, USA) with an Rtx-35ms column (30 m, 0.25 mm i.d., 0.10 μm df; Restek, Bellefonte, PA, USA) coupled to a Pegasus III MS system (Leco, St Joseph, MI, USA). The temperature program was as follows: initial temperature of 80 °C held for 1 min, increased to 150 °C at 20 °C/min and held for 10 min, then increased to 230 °C at 10 °C/min and held for 15 min. Individual FAMEs were identified by comparison with authentic standards, and relative contents were calculated as the percentage of each fatty acid peak area relative to the total fatty acid peak area.

### 2.8. Statistical Analysis

Data were analyzed and graphed using GraphPad Prism version 10.4.0 (La Jolla, CA, USA). Paired data were evaluated by Student’s *t*-test. We used a one-way ANOVA for multiple comparisons. A *p*-value less than 0.05 was considered statistically significant.

## 3. Results

### 3.1. Identification and Phylogenetic Analysis of Candidate Fad Genes

**Figure 1:**
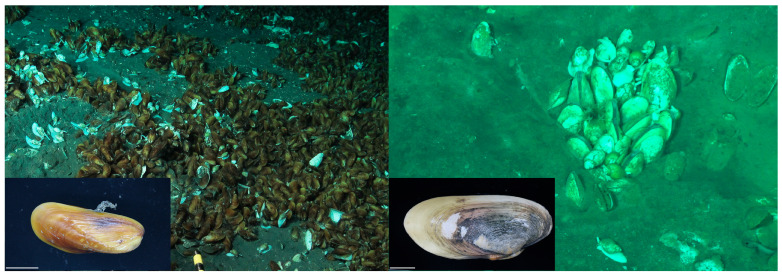
Color images of the macrofaunal ecosystem at the Haima cold seeps show beds of *G. haimaensis* and *A. marissinica*.

**Figure 2:**
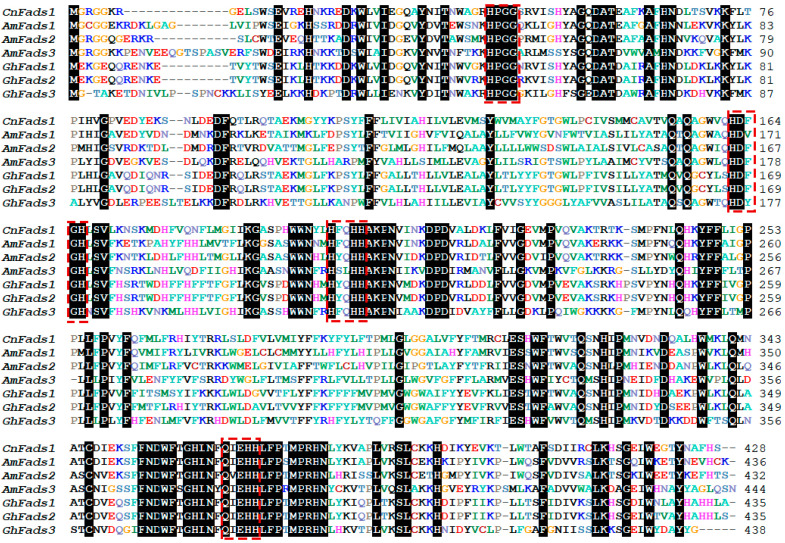
Alignment of Fad protein sequences showing conserved and variable regions. The haeme binding domain (HPGG) and the three histidine boxes (HXXXH, HXXHH, and QXXHH) are indicated with a red rectangular outline. CnFads1: Chlamys nobilis Fads1 (AIC34709.1). AmFads1, AmFads2, AmFads3: *Archivesica_marissinica* Fads. GhFads1, GhFads2, GhFads3: *Gigantidas_haimaensis* Fads.

The sampling site was characterized by active gas seepage and a thriving macrofaunal community, dominated by dense but patchy beds of the bathymodiolin mussel *G. haimaensis* and the vesicomyid clam *A. marissinica* on a muddy substrate (Figure 1). Through homology-based searches against transcriptome and genome data, we identified multiple *fad* transcripts. Both *G. haimaensis* (here named GhFads1, GhFads2, and GhFads3) and *A. marissinica* (here named AmFads1, AmFads2, and AmFads3) possessed three full-length Fads each, with lengths ranging from 432 to 444 amino acids (Table 2). These identified full-length peptides contained both the characteristic cyt-b5 (PF00173) and FA_desaturase (PF00487) domains, displaying a 47.27–61.50% similarity to the functionally characterized *C. nobilis* Fads (AIC34709). No ω3 Fad was identified in both bivalve transcriptomes and genomes, suggesting their inability to *de novo* biosynthesize the PUFA. Multiple sequence alignment of the full-length Fad proteins revealed high conservation of critical functional motifs across these Fads (Figure 2). These included the heme-binding motif (HPGG) and the three histidine-rich boxes: HXXXH, HXXHH, and QXXHH. The first histidine box conformed to the consensus sequence HD(F/V/Y)GH, with a high variation at the third amino acid. The second box (HXXHH) was variant, with a consensus of H(Y/F/S)(Q/L)HH. The final QXXHH box was a highly conserved sequence Q(I/V)EHH, with only a conservative isoleucine-to-valine substitution observed in one *A. marissinica* Fads2.

**Figure 3:**
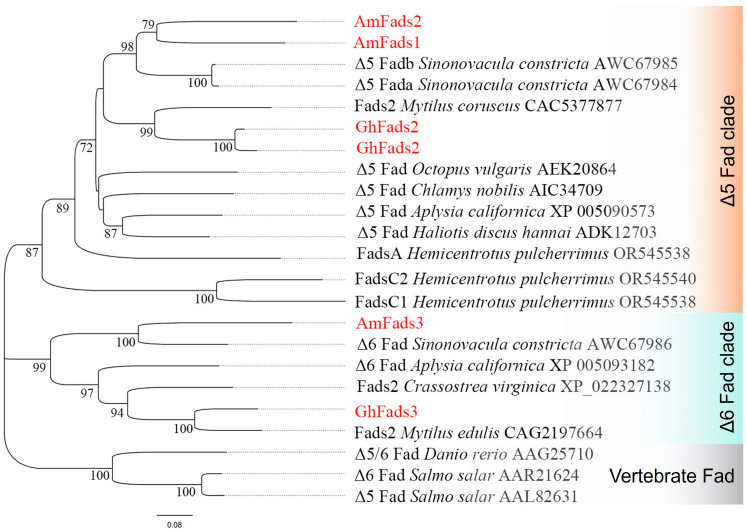
Phylogenetic tree comparing the deduced aa sequences of Fad from cold seep with their orthologues from representative vertebrates and invertebrates. The tree was constructed using the NJ method by MEGA v.11. The horizontal branch length is proportional to the aa substitution rate per site. The numbers represent the frequencies (%) with which the tree topology presented was replicated after 1000 iterations.

Fads usually form two clades according to their catalytic activity (Δ5 and Δ6/8 desaturation activity) in phylogenetic topology, which was reported both in vertebrates and invertebrates [14]. Phylogenetic analysis of the full-length ORF sequences for these cold seep Fad sequences also identified two well-supported clades (Δ5 Fad clade and Δ6/8 Fad clade) (Figure 3). Both *G. haimaensis* (GhFads1 and GhFads2) and *A. marissinica* (AmFads1 and AmFads2) had two Fads that formed distinct pairs that clustered within the Δ5 Fad clade and one Fad (GhFads3 and AmFads3) cluster within the Δ6 Fad clade, indicating a lineage-specific gene duplication event for these two species within the Δ5 Fad clade.

### 3.2. Functional Characterization of Fad Genes

**Figure 4:**
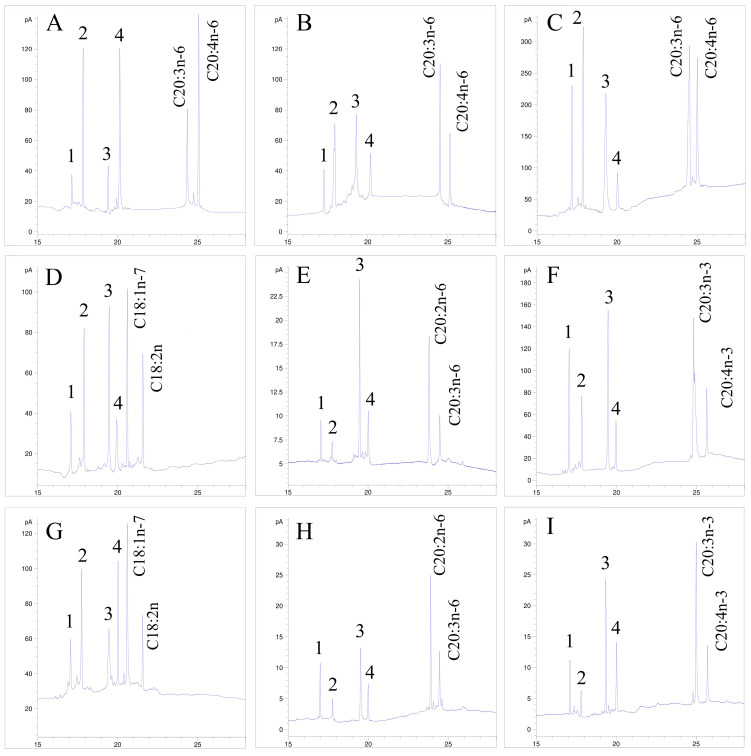
Functional characterization of the putative Fads2 from chemosymbiotic bivalves G*. haimaensis* and *A. marissinica* in transgenic yeast (*S. cerevisiae*). (**A**–**C**) FAMEs were extracted from yeast transformed with pYES2-GhFads2 (**A**), pYES2-GhFads1 (**B**), and pYES2-AmFads1 (**C**), and grown in the presence of FA substrates C20:3n-6. (**D**,**G**) FAMEs were extracted from yeast transformed with pYES2-GhFads1 (**D**) or pYES2-AmFads2 (**G**), and grown in the presence of FA substrates C18:1n-7. (**E**,**F**) FAMEs were extracted from yeast transformed with pYES2-GhFads3 in the presence of FA substrates C20:2n-6 (**E**) or C20:3n-3 (**F**). (**H**,**I**) FAMEs were extracted from yeast transformed with pYES2- AmFads3 in the presence of FA substrates C20:2n-6 (**H**) or C20:3n-3 (**I**). Peaks 1−4 correspond to the major endogenous yeast fatty acids 16:0, 16:1n-7, 18:0, and 18:1n-9, respectively. Only chromatograms showing new productions were displayed here. Horizontal axis: retention time. Vertical axis: FID response.

The putative Fad desaturases from these two seep bivalves were functionally characterized by heterologously expressing their cDNA open reading frames (ORFs) in yeast in the presence of Δ5 (C20:3n-6), Δ6 (C18:3n-3 and C18:2n-6), and Δ8 (C20:2n-6 and C20:3n-3) substrates, followed by analysis of the resulting fatty acid profiles. Yeast cells transformed with the empty pYES2 vector (control) exhibited a baseline fatty acid profile consisting primarily of C16:0, C16:1n-7, C18:0, and C18:1n-9 [13,14]. Yeast transformed with GhFads2 (Figure 4A), GhFads1 (Figure 4B), or AmFads1 (Figure 4C) displayed classical Δ5-desaturase activity, as evidenced by their ability to convert C20:3n-6 into C20:4n-6 (AA), with conversion rates of 31.37%, 76.55%, and 45.94%, respectively (Table 3). In contrast, AmFads2 showed no detectable Δ5-desaturase activity toward C20:3n-6. None of these genes exhibited Δ6- or Δ8-desaturase activity when tested with the corresponding substrates. Invertebrate Fads in the Δ5-clade have often been reported to act on MUFAs such as C18:1n-9 and C20:1n-9 to produce non-methylene-interrupted (NMI) fatty acids. Accordingly, we investigated whether AmFads1, AmFads2, GhFads1, and GhFads2 exhibit activity on MUFAs. Both GhFads1 and AmFads2 showed no activity on yeast endogenously produced C18:1n-9, as no C18:2 product was detected. Interestingly, in the presence of C18:1n-7, both GhFads1 and AmFads2 converted this MUFA substrate into a C18:2 fatty acid, as detected by GC-MS (Figure 4D). We do not have a standard for the presumed product, ^5,11^C18:2n-7. Since both enzymes are expected to possess Δ5 activity as they cluster into the Δ5 clade, we hypothesize that the new product is likely ^5,11^C18:2n-7, generated by introducing a double bond at the Δ5 position of C18:1n-7. Thus, GhFads1 exhibited broader substrate specificity than AmFads2, as it acted on both MUFA and PUFA substrates. In contrast, functional assays revealed no catalytic activity of GhFads2 or AmFads1 toward C18:1n-7.

When yeast transformed with AmFads3- or GhFads3-containing recombinant plasmids was grown in the presence of Δ5 (C20:3n-6), Δ6 (C18:3n-3 and C18:2n-6), or Δ8 (C20:2n-6 and C20:3n-3) substrates, both AmFads3 (Figure 4E,F) and GhFads3 (Figure 4H,I) converted the exogenously added C20:2n-6 and C20:3n-3 into C20:3n-6 and C20:4n-3, respectively, indicating that both enzymes possess Δ8-desaturation activity. For GhFads3, approximately 36.41% of C20:2n-6 and 35.95% of C20:3n-3 were desaturated to C20:3n-6 and C20:4n-3, respectively. For AmFads3, the conversion rates were 37.07% for C20:2n-6 and 28.19% for C20:3n-3 (Table 3). No additional desaturated products were detected when Δ6 (C18:3n-3 and C18:2n-6) or Δ5 (C20:3n-6) fatty acids were used as substrates, confirming that neither AmFads3 nor GhFads3 possesses Δ6- or Δ5-desaturation activity. Thus, both AmFads3 and GhFads3 display classical Δ8-desaturation activity.

### 3.3. Expression of Fad Genes

qPCR analysis revealed distinct tissue-specific expression patterns among the Fad genes. *GhFads1*, *AmFads1,* and *AmFads2* were most highly expressed in the gill, followed by the digestive gland, with low expression levels detected in the adductor muscle, foot muscle, and mantle. In contrast, *GhFads2*, *GhFads3*, and *AmFads3* exhibited the highest expression in the digestive gland and only low expression in the remaining tissues.

## 4. Discussion

Most deep-sea ecosystems are heterotrophic and rely on the downward flux of photosynthetic organic matter from surface waters. This chronic energy limitation is a fundamental force shaping deep-sea community structure and function [21]. A major exception to this paradigm occurs in chemosynthesis-based ecosystems, where chemical energy replaces solar energy to drive primary production [22,23]. The Haima cold seep is a classic example, sustaining exceptionally high biodiversity through dense beds of the chemosymbiotic bivalves *G. haimaensis* and *A. marissinica*. These two bivalves support higher trophic levels by hosting endosymbiotic bacteria that perform chemosynthesis. In contrast to surface habitats where organic matter is rich in essential PUFAs [14,24], bacterial biomass at cold seeps is characteristically PUFA-poor but enriched in bacterial-derived MUFAs such as C16:1n-7 and C18:1n-7 [25]. Consistent with this, both *G. haimaensis* and *A. marissinica* exhibit low levels of phototrophic PUFAs and high levels of bacterial MUFAs. Fads are key enzymes required for PUFA synthesis, yet little research has been conducted on seep bivalve Fads. Given the chronically low dietary PUFA supply at seeps, we asked whether these bivalves retain any capacity for LC-PUFA biosynthesis.

Through transcriptomic assembly, we identified three full-length Fad genes in each species, all possessing the typical features of Fad enzymes. These Fads clustered into the two major clades (Δ5 and Δ6/8) characteristic of invertebrates, as reported in other bivalves [14]. Gene duplication is a primary mechanism for generating genetic novelty and functional diversification [26,27]. We observed lineage-specific duplications within the Δ5-Fad clade of both *G. haimaensis* and *A. marissinica*. Comparable duplication events have been reported across mollusks (e.g., *H. discus*, *Crassostrea gigas*, *Lottia gigantea*) [10,12] and vertebrates (fish and mammals) [28], highlighting the widespread evolutionary role of Fad gene expansion. Functional characterization revealed that GhFads3 and AmFads3 (Δ6/8 clade) exhibit Δ8-desaturase activity on PUFA substrates, while GhFads2 and AmFads1 display classical Δ5-desaturase activity. These enzymes together enable LC-PUFA synthesis via the alternative “Δ8 pathway”. Thus, our result confirmed that these two seep bivalves retain the capability of LC-PUFA synthesis.

Functional characterization of invertebrate desaturases has established that NMI fatty acid biosynthesis is primarily catalyzed by Δ5-desaturases [15]. For example, the scallop Δ5 Fad converts C18:1n-9, C20:3n-3, and C20:2n-6 into ^Δ5,9^C18:2, ^Δ5,11,14,17^C20:4, and ^Δ5,11,14^C20:3, respectively [13]. Similarly, sea urchin FadsA introduces a Δ5 double bond into the MUFA substrates 20:1n-9 and 20:1n-7, yielding ^Δ5,11^C20:2 and ^Δ5,13^C20:2 [15]. In our study, heterologous expression confirmed that GhFads1 and AmFads2 possess the desaturase activity specifically toward C18:1n-7. Notably, neither enzyme acted on the substrates preferred by the scallop Δ5 Fad (C18:1n-9, C20:3n-3 or C20:2n-6), demonstrating distinct regioselectivity and clear functional diversification among these two seep Δ5 Fad isoforms. Since cold-seep environments are naturally enriched in n-7 fatty acids and poor in PUFA sources, the Δ5 activity on bacterial-sourced fatty acids C18:1n-7 by both GhFads1 and AmFads2 may represent a precise nutritional and environmental adaptation. In littoral bivalves, Fad genes generally show high expression in the digestive gland and low expression in muscle tissue [13,16]. Consistent with this, qPCR analysis showed that *GhFads2*, *GhFads3*, and *AmFads3* were most highly expressed in the digestive gland, with only low expression in other tissues. In contrast, *GhFads1*, *AmFads1*, and *AmFads2* were most highly expressed in the gill, followed by the digestive gland, with low expression levels in the adductor muscle, foot muscle, and mantle. Since both *G. haimaensis* and *A. marissinica* host bacteria in their gills, where symbiont-derived nutrients can be directly transferred to the host, the high expression of Δ5 Fads in the gill may facilitate more efficient desaturation of bacterial fatty acids.

## 5. Conclusions

In conclusion, this study provides a comprehensive report of Fads in two Haima cold-seep bivalves that host chemosynthetic bacterial symbionts. Our results demonstrate that these bivalves retain a high capacity for LC-PUFA biosynthesis, despite inhabiting an environment persistently deficient in PUFAs. Notably, the identified Fads exhibited desaturase activity toward bacterial C18 MUFA substrates, which constitutes a precise nutritional and environmental adaptation in cold seep bivalves.

## Figures and Tables

**Table 1:** Primer list.

Gene	Sequence (5′ to 3′)	Usage
Cox1	LCO1490: GGTCAACAAATCATAAAGATATTGG HCO2198: TAAACTTCAGGGTGACCAAAAAATCA	Cox1 fragment
GhFads1	F: CGGGGTACCCTTCCGATAAGTAGGCCTATGCAAT (Kpn I) R: TGCTCTAGAATTTAATGAATCTTGTCTGTCTTTA (Xba I)	Heterologous expression
GhFads2	F: CGGGGTACCAATTTGAAACCTGGATTGAAATGGA (Kpn I) R: TGCTCTAGATAAGTCTAATTAATAGACAAGTCAT (Xba I)	Heterologous expression
GhFads3	F: CGGGGTACCAATTTGAAACCTGGATTGAAATGGA (Kpn I) R: TGCTCTAGAACTAGTGTGTGGAGACCATGATATA (Xba I)	Heterologous expression
AmFads1	F: CGGGGTACCAATCTGAGTACTGTCGTCTGATGGG (Kpn I) R: TGCTCTAGAGATCGTCCGTGTGTAATGATGTAGG (Xba I)	Heterologous expression
AmFads2	F: CGGGGTACCACATCCACCGAATACCACTGAACAA (Kpn I) R: TGCTCTAGATGTGTTGATTCAATGTAGACTTTAT (Xba I)	Heterologous expression
AmFads3	F: CGGGGTACCACATTAGATCGTGCACTTACAAATA (Kpn I) R: TGCTCTAGAACATTACGATCACTCTCTGTCCCAC (Xba I)	Heterologous expression
GhFads1	qPCR_F: AGTCACTGGTTTGTTTGGG qPCR_R: CGTTGAAGATTCCTTGGTC	qPCR
GhFads2	qPCR_F: AGCATCCTTCTGTCCCCTA qPCR_R: GCATCCAGCCATAACTTTC	qPCR
GhFads3	qPCR_F: AGAAAAAGTTATGGCTGGA qPCR_R: AACCTCGTAGTAGAAGATGG	qPCR
AmFads1	F: TTTGATGGTTACTTTTTTGAA R: AATATTTGTGTTGTTGGTTGA	qPCR
AmFads2	F: ATTCCCGTTCAGGTAGCCA R: CAGTTCCATCCACTTTTTTC	qPCR
AmFads3	qPCR_F: TTATAATTGGACACATAAAGGG qPCR_R: TTGGTAATCATAGAGCAGGG	qPCR

Note: The underlined sequence indicates the restriction enzyme site.

**Table 2:** List of candidate genes that encode putative Fad proteins identified from transcriptome assemblies.

Species	Contig Name	Full Length	Protein Length	Pfam Domain
*Archivesica_marissinica*	AmFads2	Yes	432	cyt-b5, FA_desaturase
	AmFads1	Yes	436	cyt-b5, FA_desaturase
	AmFads3	Yes	444	cyt-b5, FA_desaturase
*Gigantidas_haimaensis*	GhFads1	Yes	435	cyt-b5, FA_desaturase
	GhFads2	Yes	435	cyt-b5, FA_desaturase
	GhFads3	Yes	438	cyt-b5, FA_desaturase

**Table 3:** Functional characterization of the putative desaturase cDNA from chemosymbiotic bivalves *G. haimaensis* and *A. marissinica* in the yeast *S. cerevisiae* cell.

Fad Gene	Substrate	Production	Conversion Ratio (%)	Activity
*GhFads1*	C20:3n-6	C20:4n-6	31.37	Δ5
	C18:2n-6	-	-	Δ6
	C20:2n-6	-	-	Δ8
	C18:1n-7	C18:2n	43.81	Δ5
*GhFads2*	C20:3n-6	C20:4n-6	76.55	Δ5
	C18:2n-6	-	-	Δ6
	C20:2n-6	-	-	Δ8
	C18:1n-7	-	-	Δ5
*AmFads1*	C20:3n-6	C20:4n-6	45.94	Δ5
	C18:2n-6	-	-	Δ6
	C20:2n-6	-	-	Δ8
	C18:1n-7	-	-	Δ5
*AmFads2*	C20:3n-6	-	-	Δ5
	C18:2n-6	-	-	Δ6
	C20:2n-6	-	-	Δ8
	C18:1n-7	C18:2n	27.94	Δ5
*GhFads3*	C20:2n-6	C20:3n-6	36.41	Δ8
	C20:3n-3	C20:4n-3	35.95	Δ8
	C18:2n-6	-	-	-
	C20:3n-6	-	-	-
	C18:1n-7	-	-	-
*AmFads3*	C20:2n-6	C20:3n-6	37.07	Δ8
	C20:3n-3	C20:4n-3	28.19	Δ8
	C18:2n-6	-	-	Δ6
	C20:3n-6	-	-	Δ5
	C18:1n-7	-	-	Δ6/8

## Data Availability

The original contributions presented in this study are included in the article. Further inquiries can be directed to the corresponding authors.
